# Mucoepidermoid carcinoma of the parotid gland with invasion of the jugular foramen region: A case report

**DOI:** 10.1097/MD.0000000000041925

**Published:** 2025-04-18

**Authors:** Yongtuan Li, Ying Wang, Hai Zhao

**Affiliations:** a Department of Otolaryngology Head and Neck Surgery, Qingdao Hospital, University of Health and Rehabilitation Sciences (Qingdao Municipal Hospital), Qingdao, China.

**Keywords:** jugular foramen region, mucoepidermoid carcinoma, parotid gland

## Abstract

**Rationale:**

Mucoepidermoid carcinoma of the parotid gland, being the most prevalent malignant neoplasm among salivary gland tumors, represents the leading incidence rate among primary parotid malignancies. This pathological entity predominantly affects young and middle-aged females, typically presenting as a painless parotid mass. Due to its variable disease progression and nonspecific clinical manifestations, preoperative misdiagnosis as a benign tumor is frequently encountered. However, when parotid mucoepidermoid carcinoma progresses to involve the jugular foramen region, a complex anatomical structure, the diagnostic and therapeutic challenges are significantly amplified.

**Patient concerns:**

This case report presents a 56-year-old male patient with difficulties in closing his right eye and deviation of the mouth corner for 1 year, along with coughing while drinking water and hoarseness for 2 months. After multiple misdiagnoses and ineffective treatments, the patient was admitted to our hospital with a suspected diagnosis of jugular foramen syndrome.

**Diagnoses:**

Radiological examinations revealed abnormal enhancement in the right parotid gland, cavernous sinus, and petrous bone of the right temporal bone, with surrounding bone destruction. Surgical pathology confirmed the diagnosis of mucoepidermoid carcinoma of the parotid gland, with intermediate grade and nerve invasion. The tumor had also invaded the jugular foramen region.

**Interventions:**

The patient underwent skull base lesion resection via the infratemporal fossa approach, parotidectomy, cervical lymph node dissection, abdominal fat filling, and external auditory canal closure. And he was treated with postoperative radiotherapy and chemotherapy.

**Outcomes:**

After 1 year and 4 months of follow-up, there was no recurrence of the tumor. The patient presented with significant deglutition-related aspiration, prompting surgical intervention to enhance quality of life. Following vocal cord augmentation performed in our department, the patient demonstrated marked improvement in both aspiration symptoms and vocal quality.

**Lessons:**

This case highlights the importance of detailed differential diagnosis and a comprehensive understanding of the jugular foramen region in avoiding misdiagnosis and ensuring timely treatment.

## 1. Introduction

Mucoepidermoid carcinoma (MEC) of the parotid gland stands as the leading malignancy within salivary gland tumors, particularly prevalent among middle-aged and young female patients. The majority of cases present with a painless parotid mass, which, coupled with its variable disease duration and nonspecific clinical manifestations, frequently leads to preoperative misdiagnosis as a benign lesion. The complexity escalates when MEC of the parotid gland advances to invade the jugular foramen, a critical anatomical region housing vital neurovascular structures. Such involvement not only complicates the diagnostic process but also poses substantial therapeutic challenges.

This report details the case of a patient who experienced a 1-year journey of misdiagnosis, ultimately confirmed with MEC of the parotid gland that had infiltrated the jugular foramen. The following narrative aims to highlight the diagnostic odyssey and the subsequent management of this rare and challenging presentation.

## 2. Case presentation

The patient, a 56-year-old male, presented with difficulties in closing his right eye and deviation of the mouth corner to the left for 1 year, with progressive worsening and facial numbness. He consulted local hospitals and underwent cranial CT and MR scans, which showed no abnormalities. He was diagnosed with peripheral facial paralysis and received neurotrophic treatment, but the effect was poor. He then sought treatment at a local traditional Chinese medicine hospital and underwent acupuncture and herbal medicine, but his symptoms did not improve. Two months ago, he developed coughing while drinking water, accompanied by hoarseness and loss of taste, with decreased sensation in the pharynx. Acupuncture treatment was ineffective, and cranial computed tomography (CT) and magnetic resonance imaging (MR) scans at a local hospital showed no abnormalities. Electronic fibrolaryngoscopy showed fixation of the right vocal cord. The patient was admitted to our hospital and diagnosed with jugular foramen syndrome.

**Figure 1.**
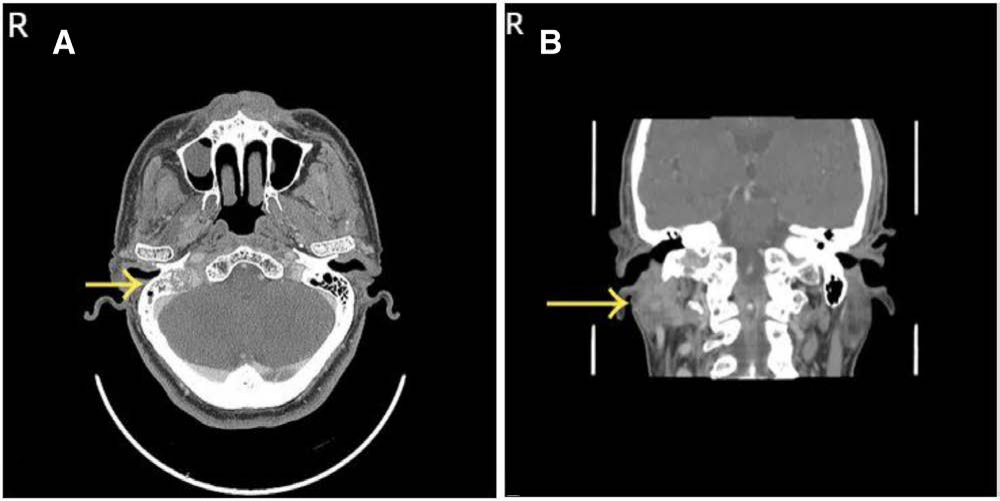
(A, B) Enhanced CT of temporal bone showed the petrous cone of the right temporal bone showed irregular enhancement density and osteolytic destruction. CT = xxx.

**Figure 2.**
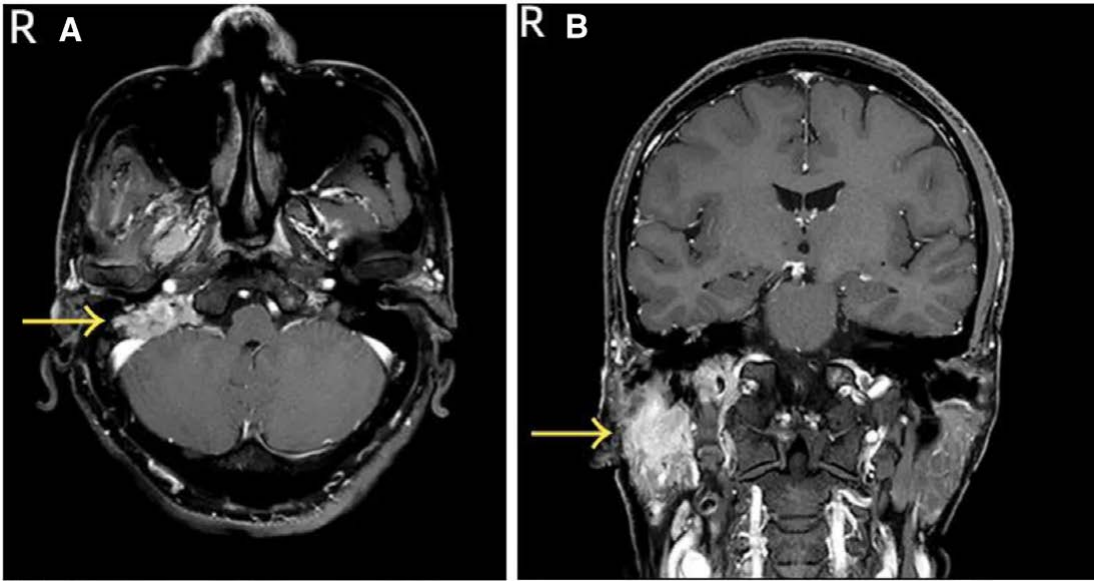
(A, B) Enhanced MR of skull base showed the right parotid gland and cerebellopontine. Angle area showed irregular T1 signals with unclear boundaries. MR = xxx.

**Figure 3.**
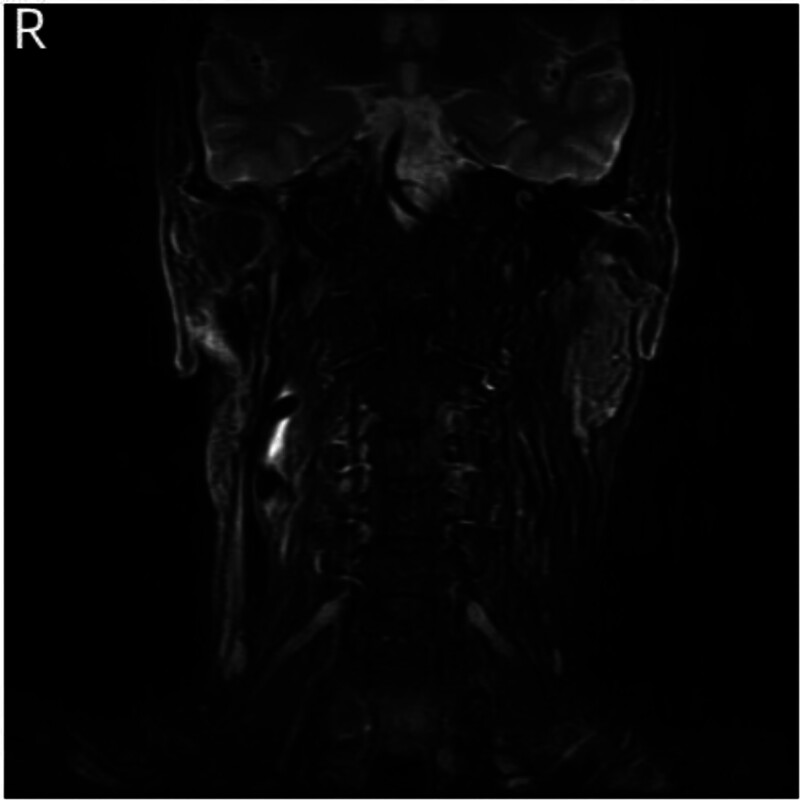
Temporal bone MR showed postoperative changes in the right parotid gland and temporal bone without tumor recurrence. MR = xxx.

Specialist examination revealed incomplete closure of the right eyelid, shallow right nasolabial fold, deviation of the mouth corner to the left, disappearance of right forehead wrinkles, and inability to whistle. The bilateral external auditory canals were patent, with intact tympanic membranes. No significant mass was palpable under the right ear. The right vocal cord was fixed, while the left vocal cord moved well with a gap in closure. Enhanced CT of temporal bone showed abnormal enhancement in the right parotid gland, cavernous sinus, and right petrous bone, with unclear boundaries and surrounding bone destruction (Fig. 1A, B). Enhanced MR of skull base showed iso-T1 signals in the medial part of the right parotid gland, with unclear boundaries, invading the right cavernous sinus anteriorly and the right cerebellopontine angle posteriorly, with uniform enhancement after enhancement (Fig. 2A, B). Based on the patient’s history, physical examination, and radiological findings, the preliminary diagnoses were: right parotid mass, right jugular foramen region occupation, jugular foramen syndrome, peripheral facial paralysis, and right vocal cord paralysis. The patient underwent skull base lesion resection via the infratemporal fossa approach, parotidectomy, cervical lymph node dissection, abdominal fat filling, and external auditory canal closure. Intraoperatively, a pale red mass was found in the right jugular foramen region, enveloping the vertical segment of the facial nerve. Multiple enlarged lymph nodes were found in the right parotid gland, with hard deep lobe tissue adhering severely to surrounding tissues. Postoperative pathology confirmed MEC of the parotid gland, intermediate grade, with nerve invasion. The mass in the jugular foramen region was infiltrating carcinoma, considered to be of parotid MEC origin. Immunohistochemistry: S100 (−), CK5/6 (−), CK7 (+), CD117 (−), DOG-1(−), SMA (−), EMA (+), Ki67 (5%+), SOX-10 (−), GFAP (−), PHH3 (nuclear fission as 0–1/ HPF), calponin (−), p40 (weakly +), p63 (−). The results of special staining were AB-PAS (+) and DPAS (−). Antibiotics were administered for 7 days postoperatively. Sutures in the ear and neck were removed 7 days after surgery, and the wound healed well. The patient underwent radiotherapy and chemotherapy in the oncology department postoperatively. After 1 year and 4 months of follow-up, there was no recurrence of the tumor (Fig. 3). The patient remains under active clinical surveillance. Due to severe deglutition-related aspiration compromising normal alimentation, the patient underwent right vocal cord augmentation with autologous rectus abdominis fascia and fat injection under laryngoscopy in our department to improve quality of life. Postoperative evaluation revealed significant improvement in both aspiration symptoms and vocal quality.

## 3. Discussion

MEC is one of the most common malignancies of the salivary glands, accounting for 8% to 12% of all salivary gland tumors and 30% to 40% of salivary gland malignancies.^[1]^ The incidence of MEC peaks between 15 and 86 years, with an average age of 49 years, and a higher incidence in females than in males.^[2]^ MEC usually occurs in the parotid gland, followed by the submandibular and sublingual glands, with good prognosis. The 5-year survival rate is approximately 98.8% for low-grade tumors, 97.4% for intermediate-grade tumors, and 67% for high-grade tumors.^[3]^ The clinical manifestations of MEC sometimes resemble those of pleomorphic adenoma, presenting as painless compressive masses with slow growth.^[4]^ The primary treatment for MEC is surgical resection, with adjuvant radiotherapy recommended in cases of perineural infiltration, lymph node involvement, high-grade tumors, positive margins after resection, and extracapsular extension.^[5]^

In this clinical scenario, the patient initially presented with facial paralysis as their primary complaint and underwent an evaluation by the attending physician. However, the physician only conducted a cranial examination to exclude the possibility of central lesions, neglecting the potential for peripheral lesions. Consequently, the patient was misdiagnosed with “Bell’s palsy.” Despite visiting multiple hospitals, all of which treated the patient based on this initial misdiagnosis, the true underlying condition remained undetected. As the patient’s condition worsened, additional symptoms such as hoarseness, dysphagia, and hypaesthesia of the pharynx emerged. Despite ongoing consultations and further examinations at local hospitals, a definitive diagnosis was never established. This repeated misdiagnosis and missed diagnosis were primarily due to a lack of clear understanding of the disease and inadequate differential diagnosis. Facial paralysis is broadly classified into central and peripheral types. Central facial paralysis arises when the lesion is located above the facial nucleus, whereas peripheral facial paralysis occurs when the lesion is within or below the facial nucleus.^[6]^ Among the various causes of, Bell’s palsy and Hunt syndrome are the most prevalent.^[7]^ In this case, the initial misdiagnosis of “Bell’s palsy” was a result of the attending physician’s failure to conduct a thorough differential diagnosis. In reality, the patient was later diagnosed with MEC of the parotid gland with invasion of the jugular foramen region, presenting as jugular foramen syndrome. A review of jugular foramen syndrome is crucial here. The jugular foramen region is a vital structure of the lateral skull base, situated between the temporal bone and occipital bone. It boasts complex anatomical relationships and houses important nerves and blood vessels.^[8]^ refers to the paralysis of ipsilateral cranial nerves IX, X, and XI due to lesions affecting the surrounding tissues or structures of 1 jugular foramen, leading to corresponding neurofunctional symptoms.^[9]^ Otolaryngologists may be less familiar with the anatomy of the jugular foramen region and jugular foramen syndrome, which can lead to oversight or misdiagnosis when encountering related cases. Therefore, otolaryngologists must possess a comprehensive understanding and mastery of the 12 cranial nerves, particularly the glossopharyngeal nerve, vagus nerve, and accessory nerve, which are closely associated with the head and neck region.

## 4. Conclusion

MEC of the parotid gland constitutes a frequently encountered clinical entity in head and neck oncology practice. The involvement of the jugular foramen region by this neoplasm presents particular diagnostic challenges due to its heterogeneous clinical presentation, often resulting in diagnostic misinterpretation and oversight, which inevitably compromises timely therapeutic intervention. The lessons learned from this case include: For patients presenting with “facial nerve paralysis,” detailed differential diagnosis is required to distinguish between central and peripheral facial paralysis and further clarify the cause of facial paralysis. Otolaryngologists should have a good understanding of the clinical knowledge related to the jugular foramen region and master the glossopharyngeal nerve, vagus nerve, and accessory nerve related to the head and neck. Although the present investigation is structured as a case report, augmentation of the sample size would provide more robust clinical evidence and substantially improve the study’s translational value in clinical practice.

## Author contributions

**Data curation:** Yongtuan Li, Ying Wang, Hai Zhao.

**Investigation:** Ying Wang.

**Writing– original draft:** Yongtuan Li, Hai Zhao.

**Writing – review & editing:** Hai Zhao.

## References

[1] Sood N Meena S Gupta R Gupta S. Histologic grading of salivary gland mucoepidermoid carcinoma: a comparison of four grading systems and correlation with survival. J Cancer Res Ther. 2024;20:57–61. 38554299 10.4103/jcrt.jcrt_1341_22

[2] Sama S Komiya T Guddati AK. Advances in the treatment of mucoepidermoid carcinoma. World J Oncol. 2022;13:1–7. 35317327 10.14740/wjon1412 PMC8913015

[3] Whaley RD Gupta S Erickson LA. Mucoepidermoid carcinoma. Mayo Clin Proc. 2023;98:1427–8. 37661153 10.1016/j.mayocp.2023.07.018

[4] Peraza A Gómez R Beltran J Amarista FJ. Mucoepidermoid carcinoma. An update and review of the literature. J Stomatol Oral Maxillofac Surg. 2020;121:713–20. 32565266 10.1016/j.jormas.2020.06.003

[5] Hsieh RC Chou YC Hung CY. A multicenter retrospective analysis of patients with salivary gland carcinoma treated with postoperative radiotherapy alone or chemoradiotherapy. Radiother Oncol. 2023;188:109891. 37659659 10.1016/j.radonc.2023.109891

[6] Devriese P Facial nerve paralysis. Ned Tijdschr Geneeskd. 1987;131:721–5. 3587388

[7] Santos-Lasaosa S Pascual-Millán LF Tejero-Juste C Morales-Asín F. Peripheral facial paralysis: etiology, diagnosis and treatment. Rev Neurol. 2000;30:1048–53. 10904952

[8] Liu J Pinheiro-Neto CD Yang D. Comparison of endoscopic endonasal approach and lateral microsurgical infratemporal fossa approach to the jugular foramen: an anatomical study. J Neurol Surg B Skull Base. 2022;83(Suppl 2):e474–83. 35832999 10.1055/s-0041-1731034 PMC9272292

[9] Das JM Al Khalili Y. Jugular Foramen Syndrome. In: StatPearls. Treasure Island (FL): StatPearls Publishing Copyright © 2024, StatPearls Publishing LLC.; 2024.

